# Elevation of neural injury markers in patients with neurologic sequelae after hospitalization for SARS-CoV-2 infection

**DOI:** 10.1016/j.isci.2022.104833

**Published:** 2022-08-01

**Authors:** Michail Spanos, Sigal Shachar, Thadryan Sweeney, H. Immo Lehmann, Priyanka Gokulnath, Guoping Li, George B. Sigal, Rajini Nagaraj, Pradeepthi Bathala, Farhan Rana, Ravi V. Shah, David A. Routenberg, Saumya Das

**Affiliations:** 1 Cardiovascular Research Center, 185 Cambridge Street, Simches 3 Massachusetts General Hospital, Boston, MA, USA; 2 Meso Scale Diagnostics, LLC. (MSD), Rockville, MD, USA

**Keywords:** Immunology, Neuroscience

## Abstract

Patients with SARS-CoV-2 infection (COVID-19) risk developing long-term neurologic symptoms after infection. Here, we identify biomarkers associated with neurologic sequelae one year after hospitalization for SARS-CoV-2 infection. SARS-CoV-2-positive patients were followed using post-SARS-CoV-2 online questionnaires and virtual visits. Hospitalized adults from the pre-SARS-CoV-2 era served as historical controls. 40% of hospitalized patients develop neurological sequelae in the year after recovery from acute COVID-19 infection. Age, disease severity, and COVID-19 infection itself was associated with additional risk for neurological sequelae in our cohorts. Glial fibrillary astrocytic protein (GFAP) and neurofilament light chain (NF-L) were significantly elevated in SARS-CoV-2 infection. After adjusting for age, sex, and disease severity, GFAP and NF-L remained significantly associated with longer term neurological symptoms in patients with SARS-CoV-2 infection. GFAP and NF-L warrant exploration as biomarkers for long-term neurologic complications after SARS-CoV-2 infection.

## Highlights

• 40% of hospitalized patients with COVID-19 developed neurological sequelae after recovery
• Age, disease severity, and COVID-19 infection were associated with neurological sequelae
• GFAP and NF-L were significantly elevated in SARS-CoV-2 infection compared to controls
• GFAP and NF-L were elevated in patients with long-term neurological symptoms

## Graphical abstract

**Figure:**
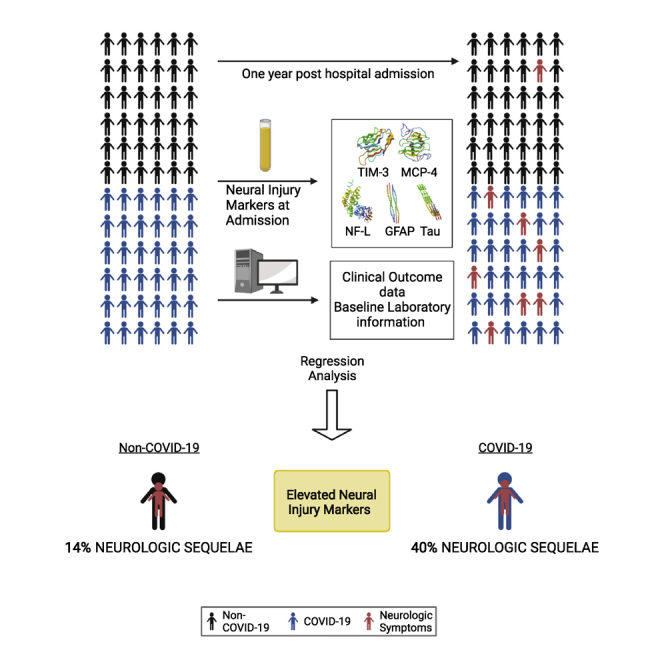

## Introduction

The coronavirus disease 2019 (COVID-19) is increasingly recognized as a multi-organ disease that extends beyond the respiratory system (Thakur et al., 2021b). In the majority of cases, symptoms of the illness subside three to seven days after onset (Dinh et al., 2021). However, reports of neurologic, pulmonary, cardiac, and gastrointestinal symptoms that persist, 14 to 110 days after recovery, have been described as a “post-acute COVID-19 syndrome” (Lopez-Leon et al., 2021). “Post-acute COVID-19 syndrome” is thought to occur in up to 80% of patients that had been infected with the severe acute respiratory syndrome coronavirus 2 (SARS-CoV-2) (Mo et al., 2020; Jha et al., 2021; Lopez-Leon et al., 2021; Peluso et al., 2021; Spotnitz et al., 2021). Indeed, a variety of neuropsychiatric symptoms have been reported at around 6 months after an index hospitalization for COVID-19 in up to 82%–90% of patients (Koralnik and Tyler, 2020), leading to long-term decrease in functional capacity and adverse cognitive outcomes (Frontera et al., 2021). Most commonly reported symptoms for the “post-acute COVID-19 syndrome” have been fatigue, loss of smell, concentration, short-term memory deficits (“brain fog”), and anxiety (Logue et al., 2021). Recent studies have shown increased levels of neuroinflammatory proteins and neural injury markers such as glial fibrillary acidic protein (GFAP), neurofilament light chain (NFL), and Tau in patients with COVID-19 in plasma or cerebrospinal fluid (CSF) (Frithiof et al., 2021). Here, we hypothesized that plasma neuronal injury and neuroinflammation markers in the plasma may reflect pathological processes in the brain, and are associated with long-term neurological deficits in patients who overcame SARS-CoV-2 infection.

## Results

### Study population

Baseline characteristics of the study population are shown in Table 1. Our final analytic sample consisted of 61 patients with COVID-19. Eight patients (13%) had early in-hospital fatal outcomes and thus were excluded from all further analysis. 21 (40%) patients developed neurologic symptoms within one year after the recovery, of which 20 were from the hospitalized cohort, and only 1 was from the outpatient cohort. 14 patients had more than one neurological symptoms. More specifically, 14 patients developed cognitive impairment, 7 patients developed CNS symptoms, 7 patients manifested PNS symptoms, and 9 patients showed signs of musculoskeletal weakness. Of the patients with neurological symptoms, the majority (16 patients, 76%) had received ICU level of care.

**Table 1:** COVID-positive patients and controls

	Overall COVID, n = 61	Hospitalized COVID = 48	Hospitalized non-COVID controls, n = 60	*p*
Hospitalized, n (%)	48/61 (78.6)	48/48 (100)	60/60 (100)	
Age, mean (SD), years	54.9 (15)	64.5 (13)	64 (16)	0.119
Sex, male n (%)	35/61 (51)	19/48 (39.5)	35/60 (58)	1.00
Smoking, n (%)	17/61 (27.8)	14/48 (29)	NA	
Alcohol, n (%)	26/61 (42.6)	13/48 (27)	NA	
BMI, mean (SD), Kg/m^2^	32 (8)	32.7 (7.5)	NA	
Heart rate, mean (SD), beats/min	86 (17)	86 (19)	87 (19)	0.725
Respiration, mean (SD), resp/min	23.38 (7.3)	26 (7)	19 (2)	<0.001
Hospitalization duration, mean (SD), days	28 (22.16)	28 (22.16)	14.2 (15)	<0.001
ICU Duration, mean (SD), days		39.6 (17)	21.8 (20)	<0.001
BUN, mean (SD), mg/dL	22 (16)	28 (19)	35 (22)	0.152
Creatinine, mean (SD), mg/dL	1.3 (1.2)	1.4 (1.5)	1.6 (0.8)	0.611
eGFR, mean (SD), mL/min	75 (33)	74 (34)	48 (25)	<0.001
Alkaline Phosphatase, mean (SD), IU/L	113 (125)	139.6 (158.4)	113.4 (80.0)	0.315
AST, mean (SD)	51.8 (52.9)	61.8 (57.1)	40.8 (30.5)	0.029
ALT, mean (SD)	47.6 (41.3)	52.8 (46.1)	45.4 (67.4)	0.510
AST/ALT, mean	1.2 (0.6)	64 (55)	1.27 (0.51)	0.320
Albumin, mean (SD), mg/dL	3 (0.7)	2.9 (0.5)	NA	
WBC Count, mean (SD), K/μL	9.4 (4.1)	10.0 (4.4)	9.2 (3.9)	0.315
RBC, mean (SD), M/μL	4.1 (0.9)	3.9 (0.9)	4.2 (1.1)	0.107
Hb, mean (SD), g/dL	12.7 (2.5)	11.0 (2.1)	12.2 (3.6)	0.046
Neutrophils, mean (SD), %	70 (14)	72.8 (12.8)	72.3 (9.0)	0.653
Immature Granulocytes, mean (SD), %	0.9 (0.9)	1.4 (1.2)	NA	
Lymphocytes, mean (SD), %	20.8 (11)	15.6 (11.1)	16.7 (7.9)	0.201
N/L Ratio, mean (SD)	6 (5)	9.4 (14.2)	6.0 (5.3)	0.143
CRP, mean (SD), mg/L	74 (88)	112.5 (96.2)	53.3 (69.8)	0.006
ESR, mean (SD), mm/h	50 (49)	68.5 (44.7)	40.1 (28.6)	0.007
Procalcitonin, mean (SD), ng/mL	0.7 (2.7)	1.69 (4.4)	NA	
Ferritin, mean (SD), μg/L	574 (520)	1145.1 (2627.2)	176.9 (184.4)	0.019
D-dimer, mean (SD), ng/mL	1957 (2299)	8995 (2166)	NA	
Qsofa >1, n (%)	16/61 (18)	16/45 (35)	11/49 (14)	0.178
WHO, Mild, n (%)	15/61 (24.5)			
WHO, Moderate, n (%)	16/61 (26.2)	16/48		
WHO, Severe, n (%)	22/61 (36)	30/48		
WHO, Fatal, n (%)	8/61 (13)^a^	8/53^a^		

COVID-19, Coronavirus Disease; BMI, Body Mass Index; ICU, Intensive Care Unit; BUN, Blood Urea Nitrogen; eGFR, Glomerular Filtration rate (CKD-EPI equation); WBC, White Blood Cell, RBC, Red Blood Cell; Hb, Hemoglobin; N/L, Neutrophil/Lymphocyte ratio; CRP, C-Reactive Protein; ESR, Erythrocyte Sedimentation Rate; WHO, World Health Organization; WHO (mild, moderate, severe, fatal), World Health Organization COVID19 ordinal scale.

All measurements were made in the hospital setting. Data are shown as n, (%) or mean ± Standard Deviation.

Bold values indicate a statistically significant difference with a p value < 0.05.

COVID-19, Coronavirus Disease; BMI, Body Mass Index; ICU, Intensive Care Unit; BUN, Blood Urea Nitrogen; eGFR, Glomerular Filtration rate (CKD-EPI equation); WBC, White Blood Cell, RBC, Red Blood Cell; Hb, Hemoglobin; N/L, Neutrophil/Lymphocyte ratio; CRP, C-Reactive Protein; ESR, Erythrocyte Sedimentation Rate; WHO, World Health Organization; WHO (mild, moderate, severe, fatal), World Health Organization COVID19 ordinal scale.

All measurements were made in the hospital setting. Data are shown as n, (%) or mean ± Standard Deviation.

Bold values indicate a statistically significant difference with a p value < 0.05.

a Fatal cases were excluded from Follow-up and further analyses and are not included in the neurological sequelae section of the table.

Within the control population without COVID-19 infection but matched for age, sex, and disease severity (n = 60), follow-up data were available in 47 patients (78%), of whom 7 (14%) developed neurologic symptoms within one year after hospitalization. Of those, 3 patients had received ICU level treatment and 4 were treated in a non-ICU setting. Six of the patients that developed neurologic symptoms and 2 patients from the non-neurologic group had undergone brain MRI.

### Clinical characteristics of patients with neurological sequelae

Patients with COVID-19 had a longer time of ICU care (COVID-19; 39.6 days, non-COVID-19; 21.8 days p < 0.001), exhibited lower hemoglobin values and significantly increased levels of C-reactive protein, erythrocyte sedimentation rate, and Ferritin, compared to non-COVID-19 Controls consistent with increased inflammation and immune response (Table 1).

Neurological events in both cohorts were associated with longer hospitalization and increased disease severity. Within the COVID-19 cohort, compared to patients without neurological symptoms, patients with neurologic sequelae were older and exhibited multi-organ dysfunction, manifested pulmonary disease, signs of hepatic injury, and decreased renal function. Moreover, patients with neurological sequelae had significant leukocytosis, lymphopenia, and greatly elevated neutrophil-to-lymphocyte ratio (N/L; 8.4, p = 0.015). Inflammatory markers were consistently elevated correlating to greater disease severity (Table 2). Of note, MRI studies of the neurological patients demonstrated sparse microhemorrhages, foci of T2/fluid-attenuated inversion recovery (FLAIR) signal abnormalities, and diffusion restriction within the subcortical and periventricular white matter, corpus callosum, and cerebellar white matter with peripheral normal to slightly elevated diffusion. MRI studies of patients from the non-neurologic group were normal. Within the control cohort, ICU duration was associated with persistent neurologic symptoms, whereas liver, renal function, or age were not.

**Table 2:** COVID-positive patients with and without neurological sequelae

	COVID Non-neurological sequelae, n = 32	COVID neurological sequelae,^a^ n = 21	*p*
Hospitalized, n (%)	21/32 (65.6)	20/21 (95)	
Age, mean (SD), years	46 (15)	61 (13)	**<0.001**
Sex, male n (%)	23/32 (71.8)	12 (57)	0.717
Smoking, n (%)	11 (23.4)	8 (38.1)	0.276
Alcohol, n (%)	20/32 (62.5)	6/21 (28.6)	0.544
BMI, mean (SD), Kg/m^2^	32 (9)	32 (6)	0.772
Heart rate, mean (SD), beats/min	87 (19)	85 (15)	0.753
Respiration, mean (SD), resp/min	21 (6)	26 (8)	**0.014**
MRI imaging, n (%)	2 (6)	6 (28.5)	
ICU Duration, mean (SD), days	37 (18)	45 (18)	0.341
BUN, mean (SD), mg/dL	17 (9)	32 (21)	**<0.001**
Creatinine, mean (SD), mg/dL	0.9 (0.3)	1.9 (1.9)	**0.005**
eGFR, mean (SD), mL/min	88 (26)	61 (34)	**0.008**
Alkaline Phosphatase, mean (SD), IU/L	89 (77)	158 (178)	0.051
AST, mean (SD)	36.8 (34)	50.7 (27.6)	0.112
ALT, mean (SD)	49.7 (42)	42.8 (30.9)	0.517
AST/ALT, mean	0.9 (0.5)	1.3 (0.5)	**0.004**
Albumin, mean (SD), mg/dL	3 (0.6)	2.9 (0.5)	**<0.001**
WBC Count, mean (SD), K/μL	7.8 (2.65)	10 (4.8)	0.002
RBC, mean (SD), M/μL	4.6 (0.6)	3.7 (0.9)	**<0.001**
Hb, mean (SD), g/dL	13.7 (1.99)	10.7 (2.33)	**<0.001**
Neutrophils, mean (SD), %	62 (12.4)	73.7 (13)	**0.004**
Immature Granulocytes, mean (SD), %	0.7 (0.8)	1.3 (1)	**0.032**
Lymphocytes, mean (SD), %	24 (9)	14 (11)	**0.001**
N/L Ratio, mean (SD)	4.4 (4)	8.4 (5.7)	**0.015**
CRP, mean (SD), mg/L	32.6 (46)	138 (100)	**<0.001**
ESR, mean (SD), mm/h	29 (39)	82 (46)	**<0.001**
Procalcitonin, mean (SD), ng/mL	0.1 (0.2)	1.4 (4)	0.118
Ferritin, mean (SD), μg/L	428 (388)	784 (618)	**0.017**
D-dimer, mean (SD), ng/mL	1178 (1807)	2921 (2513)	**0.008**
Qsofa >1, n (%)	3/32 (9.3)	8/21 (38)	
WHO, Mild, n (%)	14/32 (43.7)	1/21 (4.7)	
WHO, Moderate, n (%)	12/32 (37.5)	4/21 (19)	
WHO, Severe, n (%)	14/32 (43.7)	16/21 (76)	
WHO, Fatal, n (%)	NA	NA	

COVID-19, Coronavirus Disease; BMI, Body Mass Index; ICU, Intensive Care Unit; BUN, Blood Urea Nitrogen; eGFR, Glomerular Filtration rate (CKD-EPI equation); WBC, White Blood Cell, RBC, Red Blood Cell; Hb, Hemoglobin; N/L, Neutrophil/Lymphocyte ratio; CRP, C-Reactive Protein; ESR, Erythrocyte Sedimentation Rate; WHO, World Health Organization; WHO (mild, moderate, severe, fatal), World Health Organization COVID19 ordinal scale.

All measurements were made in the hospital setting. Data are shown as n, (%) or mean ± Standard Deviation.

Bold values indicate a statistically significant difference with a p value < 0.05.

a Fatal cases were excluded from follow-up and further analyses and are not included in the neurological sequelae section of the table.

### Biomarkers of brain injury and inflammation in COVID-19 or control patients

**Figure 1:**
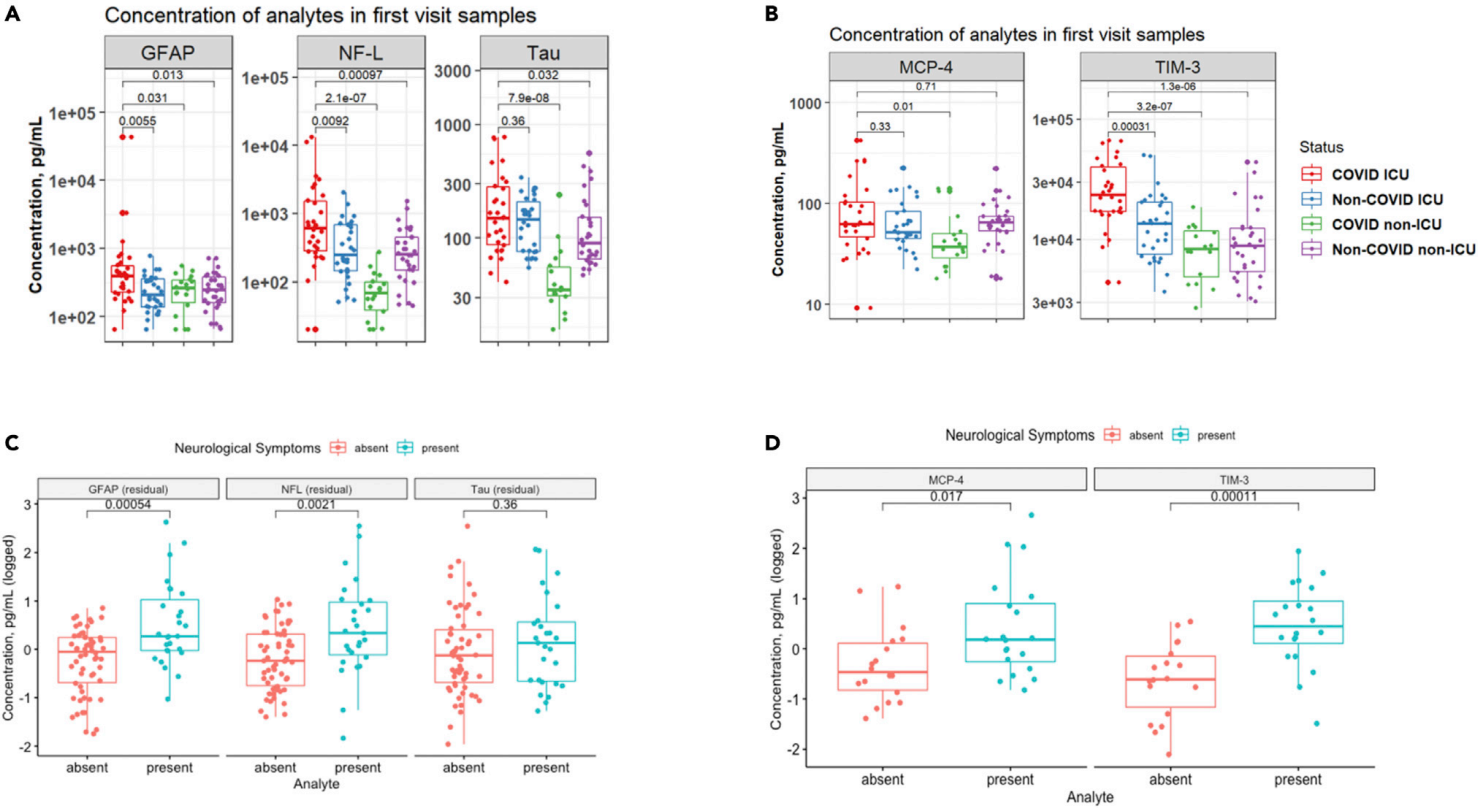
Plasma concentration levels of neuronal injury markers and microglial activators MCP-4 and TIM-3 in hospitalized patients Plasma concentration levels showing: A) Neuronal injury markers and B) microglial activators MCP-4 and TIM-3 from hospitalized patients with or without COVID. C) Neuronal injury markers from all hospitalized patients (COVID and Controls) stratified by neurological symptoms after age residualization and adjustment for age, sex, and severity. D) MCP-4 and TIM-3 from hospitalized patients with COVID stratified by neurological symptoms. The boxplots depict median and interquartile values. Wilcoxon rank-sum test was used to indicate statistical significance. A p-value <0.05 was considered as threshold.

Circulating neural injury markers GFAP and NFL levels were higher in patients with COVID-19 relative to non-COVID-19 controls in the patients receiving ICU level care (GFAP; p = 0.005, NFL; p = 0.009; Figure 1A). No significant differences were observed between the two groups when they were not treated in the ICU setting (Figure 1A). The plasma concentration of Tau was not significantly different between patients with COVID-19 and controls (Figure 1A). Tim-3 was significantly elevated in patients with COVID-19 relative to non-COVID-19 controls who were treated in the ICU setting while MCP-4 levels were not significantly different between the two groups (Figure 1B). Notably, these markers were not significantly different between uninfected controls and asymptomatic COVID-19-positive patients in the ambulatory setting (Figures S1 and S2).

### Association of biomarkers with neurological sequelae

NF-L and GFAP were significantly elevated in all hospitalized patients (COVID-19 and Controls) who developed long-term neurological symptoms after adjustment for age and disease severity (Figure 1C). While MCP-4 and TIM-3 were significantly elevated only in patients with COVID-19 who developed long-term neurological symptoms (MCP-4 p = 0.017, TIM-3 p = 0.0001; Figure 1D), these latter differences were not statistically significant when adjusted for disease severity.

**Figure 2:**
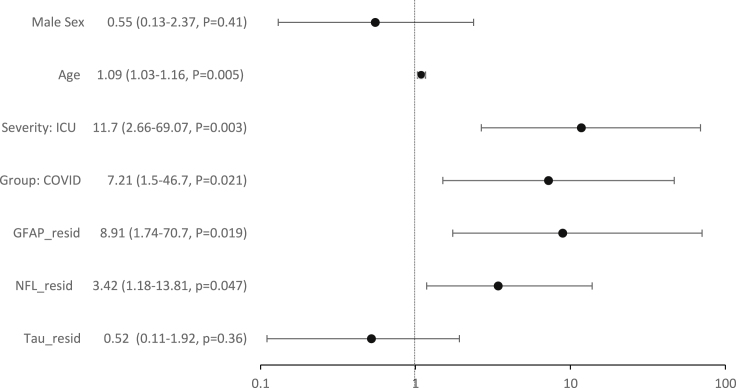
Multivariable regression analysis for neurological symptom development Forest Plot showing odds ratios and 95% confidence intervals for neuronal injury markers after age subtraction with residualization, age, severity (ICU admission), COVID-19, and male sex. The vertical line indicates an OR value of 1.

In a multivariable regression model that included sex, age, and disease severity, both disease severity and COVID-19 infection were associated with subsequent neurological sequelae (Figure 2). Notably, patients with COVID-19 had significantly higher likelihood of developing neurological symptoms within one year after hospitalization compared to non-COVID-19 controls (OR = 7.21, 95% CI: 1.5–46.7, p = 0.02; Figure 2). In our adjusted model, higher levels of GFAP and NF-L were associated with the development of neurological symptoms (GFAP: OR = 8.91, 95% CI: 1.7–70.7, p = 0.001, NF-L: OR = 3.42, 95% CI: 1.18–13.8 p = 0.04; Figure 2).

## Discussion

Even prior to the current omicron wave, the number of confirmed cases of COVID-19 mounted to 9.3 million in the UK and 46.4 million in the US with 14%–30% of hospitalized patients requiring treatment in the ICU (Grasselli et al., 2020; Huang et al., 2020; Richardson et al., 2020; Worldometers.info, 2021). Epidemiological studies suggest that a substantial number of patients recovered from acute COVID-19 infection suffer long-term sequelae, termed “long COVID” (Moreno-Pérez et al., 2021; Nune et al., 2021; Taquet et al., 2021b). Symptoms have been reported in as many as 1 in 3 patients 3–6 months after recovery from initial infection, with the risk increasing with disease severity (Taquet et al., 2021bTaquet et al., 2021b). More specifically, epidemiological studies have also noted the increased likelihood of long-term neurological sequelae in recovered patients with COVID-19, with the likelihood rising with disease severity, but the pathophysiologic mechanism remains largely unknown (Graham et al., 2021; Logue et al., 2021; Taquet et al., 2021a). In our study, more than 34% of patients infected with SARS-CoV-2 developed persisting or newly presenting neurological symptoms after disease recovery, especially those who were treated in ICU setting. By this estimation, more than 400,000 and two million ICU patients in the UK and US respectively are at risk of developing neurologic symptoms after infection. Yet, the underlying mechanism of this entity remains largely unknown, highlighting the need for both mechanistic understanding of long COVID, and biomarkers that may help with diagnosis, prognosis, and prediction of outcomes.

It remains unclear if the development of neurological sequelae is driven solely by the degree of disease severity or if there are unique characteristics specific to the SARS-CoV2 virus that play a role in the pathogenesis of this entity. While our study confirms previous findings that disease severity is associated with neurological symptoms in patients, we further show that SARS-CoV-2 infection itself is additionally associated with the development of neurological sequelae when compared to a cohort of hospitalized patients matched for age and disease severity. These findings are consistent with a prior epidemiological study that demonstrated the higher likelihood of neurological symptoms in patients with COVID-19 compared to patients infected with influenza (Taniguchi et al., 2022). Additionally, in our cohort, previously described markers of brain injury GFAP and NF-L, were elevated in patients with SARS-CoV-2 infection who developed neurologic symptoms during 1-year follow-up. While these markers may be expected to increase in older patients and patients receiving ICU care (Chatterjee et al., 2021b; Frithiof et al., 2021), our analysis (with residualization for age and disease severity) suggests association of these markers with neurological damage independent of their association with age and disease severity.

Markers of neural/glial injury and neuroinflammation have long been used in patients suffering TBI, showing diagnostic and prognostic utility (Ramlawi et al., 2006; Lumpkins et al., 2008; Shahim et al., 2017, 2020). Recently, in patients with COVID-19, astrocytic and neural injury markers have been correlated to COVID-19 severity but failed to predict subsequent long-term neurological outcomes (Kanberg et al., 2021). However, that study did not show a correlation of neurological outcome symptoms with disease severity which has been shown in several trials (Hopkins and Brett, 2005; Hopkins and Jackson, 2006; Herridge et al., 2016; Ibrahim et al., 2021). While it is clear that ICU level care and disease severity (likely associated with higher levels of peripheral inflammation and hypoxemia) in both patients with COVID-19 and pre-COVID historical patients is associated with both neurological sequelae and elevated brain injury, infection with SARS-CoV2 infection itself appears to markedly increase the likelihood of developing subsequent neurological symptoms. These findings are in line with several recent autopsy studies that have reported findings of acute hypoxic injury, hemorrhage, mild to moderate non-specific inflammation, marked microglial activation, transcriptional changes in astroglial cells, and possible neuronal damage (Matschke et al., 2020; Puelles et al., 2020; Schaller et al., 2020; Schurink et al., 2020; Solomon et al., 2020; Wichmann et al., 2020; Thakur et al., 2021a). Of note, the direct neuroinvasiveness and neurotropism of SARS-CoV-2 has now been shown in animal experiments and CNS organoids, where SARS-CoV-2 infection was shown to have detrimental effects to both infected and neighboring neurons (Ramani et al., 2020; Zhang et al., 2020; Kumari et al., 2021; Song et al., 2021). Conversely, evidence for direct SARS-CoV-2 primary infection of brain cells has been limited; two of the largest recent brain autopsy studies from COVID-19-positive patients revealed hypoxic/ischemic changes, hemorrhagic infarcts, and microglial activation with microglial nodules, most prominently in the brainstem and periventricular subcortical white matter. However, in all cases, viral RNA levels were very low to undetectable, and did not correlate with the histopathological alterations (Matschke et al., 2020; Thakur et al., 2021a). Interestingly, lesions in these areas of the brain were also confirmed in patients of our study cohort with neurological symptomatology who underwent brain MRI and exhibited scattered microhemorrhages and T2/FLAIR hyperintense foci in the subcortical and periventricular white matter of the cerebral hemispheres and parts of brainstem. In fact, this pattern of neuroimaging abnormalities has been described among the most common brain MRI parenchymal signal abnormalities that have been associated with SARS-CoV-2 (Gulko et al., 2020; Kihira et al., 2020; Kremer et al., 2020).

The brain injury biomarkers we have characterized have been studied in the context of other neurological diseases, including Alzheimer disease and traumatic brain injury (TBI) (Mondello et al., 2020; Thijssen et al., 2020; Chatterjee et al., 2021a; Cicognola et al., 2021; Clarke et al., 2021). GFAP is an astrocytic biomarker that is elevated during neuronal injury, glial activation, and scarring (Ramlawi et al., 2006; Kanberg et al., 2020; Lafrenaye et al., 2020; Frithiof et al., 2021). NF-L is an intra-axonal structural protein that was found to be elevated after TBI and is also associated with COVID-19 disease severity (Shahim et al., 2017, 2020; Kanberg et al., 2020). Interestingly, we observed that there appears to be considerable biomarker and symptom overlap between patients with TBI and post-COVID neurological sequelae. While direct viral invasion of brain parenchyma is, to date, as mentioned above, limited to the detection of low levels of viral RNA and viral antigens in cranial nerves, in patients with COVID-19, hypoxia along with neuroinflammation and microglial activation, as has been described both in TBI and more recently, in patients with COVID-19, may contribute to these effects in COVID-recovered patients (Matschke et al., 2020; Thakur et al., 2021a). In fact, microglial hyperactivation in several regions of the brain such as brainstem and hippocampus may serve as the pathological common ground between the shared observed neuropathologies in patients with COVID-19 and TBI (Donat et al., 2017; Lier et al., 2020; Poloni et al., 2021). In par with that, a recent study profiling brain tissue of patients with COVID-19 found microglial activation and increased tissue GFAP levels (Yang et al., 2021)^.^ Microglial stimulating molecules such as MCP-4 and TIM-3 are implicated in monocyte activation, neutrophilic recruitment, neuroinflammation, and microglial activation (Mendez-Enriquez and García-Zepeda, 2013; Koh et al., 2015). In our study, MCP-4 and TIM-3 were also significantly elevated in COVID-19 ICU patients who developed neurologic sequelae, although this likely reflects increased disease severity in these patients. Nonetheless, immune-mediated neuro-astroglial destruction and hypoxic neuroinflammation could be one of the underlying mechanisms for long-term neurological symptomatology.

### Limitations of study

Our analyses assessing associations of long-term neurological sequelae with serum biomarkers are hypothesis generating and have several limitations. First, our cohort of patients with SARS-CoV-2 is small and only patients who were tested in the hospital setting were focused on (as our small cohort of COVID-19-infected outpatients had only 1 patient with neurological sequelae), making our study vulnerable for selection bias. Second, as known and confirmed by our analysis, these biomarkers are significantly influenced by disease severity and age which was adjusted for using statistical modeling and residualization. Third, neurological outcomes were by physician assessment only without more detailed investigation and with the exception of 8 patients, no imaging studies were conducted. Our control group of patients without COVID-19 was not contemporaneous and the follow-up of those patients was not primarily designed to detect neurological symptoms and thus, could underreport these symptoms. Finally, the effect of vaccination and the outcome of the omicron variant (with reportedly less anosmia) would be of great interest, but not determined in this study. Further investigation of these markers in larger cohorts of patients with COVID-19 disease across the spectrum of disease severity and variants and their association with short- and long-term neurological sequelae is warranted.

## STAR★Methods

### Key resources table

**Table:** 

REAGENT or RESOURCE	SOURCE	IDENTIFIER
**Biological samples**		

COVID Plasma samples	Partners COVID biorepository	N/A

**Critical commercial assays**		

U-PLEX Effector Cell Checkpoint Combo 1 panel	Meso Scale Diagnostics, Rockville, MD USA	https://www.mesoscale.com/en/products/u-plex-chemokine-combo-1-human-k15047k/
V-PLEX Chemokine 1 panel, K15047D	Meso Scale Diagnostics, Rockville, MD USA	https://www.mesoscale.com/en/products/v-plex-chemokine-panel-1-human-kit-k15047d/
Ultrasensitive GFAP	Meso Scale Diagnostics, Rockville, MD USA	N/A
Ultrasensitive Tau	Meso Scale Diagnostics, Rockville, MD USA	N/A
Ultrasensitive NF-L	Meso Scale Diagnostics, Rockville, MD USA	N/A

**Deposited data**		

Elevation of Neural injury markers in Patients with Neurologic Sequelae after Hospitalization for SARS-CoV-2 Infection. Spanos et al	Mendeley Data	https://doi.org/10.17632/y8dfts7n7b.1

**Software and algorithms**		

R (v.4.2.1)	The R project	https://www.r-project.org/
RStudio (v.2022.02.0)	The R project	https://www.rstudio.com/products/rstudio/
SPSS	IBM Software	https://www.ibm.com/products/spss-statistics
Microsoft Excel	Microsoft Office	https://www.microsoft.com/en-us/microsoft-365

### Resource availability

#### Lead contact

Further information and requests for resources and reagents should be directed to and will be fulfilled by the lead contact, Saumya Das (sdas@mgh.harvard.edu).

#### Materials availability

This study did not generate new unique reagents.

### Experimental model and subject details

#### Ethics statement

The study was approved by institutional ethical boards, and samples from subjects enrolled in the MGB were subjected to measurement and functional assays following approval of our protocol for secondary use of research samples and data (IRB #:2020P001547, approved 5/18/2020)

#### Study design and participants

The COVID-19 cohort included 61 individuals in the Mass-CPR or main Partners COVID biorepository who presented to the Massachusetts General Hospital with suspected SARS-CoV-2 infection from 3/19/20 to 9/3/2020. A confirmed case of COVID-19 was defined as a positive result on real-time polymerase chain reaction (PCR) of throat swab specimens. Disease severity was categorized according to the World Health Organization COVID19 ordinal scale (Marshall et al., 2020). We included 60 hospitalized patients from an observational cohort study conducted from 2016 to 2019 as non-COVID-19 controls. Of those, 30 patients received ICU level of care. Patients in the non-COVID-19 control sample were matched according to age, sex, ICU duration, and quick Sepsis Related Organ Failure Assessment (qSOFA) scores.

#### Follow-up and adjudication of neurologic events

Clinical data and medical history of all patients were collected during the hospitalization. Screening of COVID-19-positive patients for neurological symptoms was conducted using an on-line self-report questionnaire, and “virtual” clinical visits during one year after hospitalization. Patients who screened positive were evaluated by a neurologist for further assessment of short-term memory, working memory, visuospatial abilities, abstract reasoning attention, concentration, and executive functions. Follow-up for patients in the control sample was conducted in routine physician visits.

Neurologic symptoms were categorized into four categories: (1) Cognitive symptoms (concentration abnormalities or “brain fog,” memory deficits); (2) central nervous system (CNS) symptoms (new history of headaches, dizziness, fatigue, seizures, impaired consciousness); (3) peripheral nervous system (PNS) symptoms (loss of smell/taste, paresthesias); (4) musculoskeletal symptoms (weakness/instability). Patients with neurological disease at baseline were excluded. Patients with fatal outcomes during hospitalization were excluded from all further analyses.

### Method details

#### Plasma handling and protein marker quantification

EDTA plasma samples were collected during admission and stored at −80°C. The concentrations of neural injury markers GFAP, NF-L and total Tau were measured in plasma samples using MSD S-PLEX ultrasensitive ECL immunoassays and the concentrations of MCP-4, TIM-3 were measured in the same samples using MSD V-PLEX and U-PLEX ECL immunoassays, respectively (Meso Scale Diagnostics, Rockville, MD USA). All assays were performed according to the manufacturer’s instructions. Samples were blinded, randomized, and diluted two-fold prior to assaying GFAP, NF-L, Tau, and MCP-4 or 100-fold prior to measuring TIM-3. All measured concentrations fell within the quantifiable range for Tau, MCP-4, and TIM-3. A few samples fell below the quantifiable range for GFAP and NF-L and for the statistical analyses the concentrations of these samples were assigned as the lower limit of detection (LLOD) for each assay.

### Quantification and statistical analysis

Univariate comparisons between biomarkers and COVID-19 status were performed using the Wilcoxon test. Logistic regression modeling was used to measure association between biomarkers and neurological outcome. Covariates in models included age, sex, disease severity (by WHO status), GFAP, NFL, and Tau. Each biomarker was residualized via linear regression against age and WHO status, given the postulated and observed collinearity between age, disease status, and neural injury markers(Vågberg et al., 2015), and biomarker residuals were entered into logistic models. Model comparison (between models with and without biomarkers) was performed by the likelihood ratio test. A p value <0.05 was considered to indicate statistical significance.

## Data Availability

De-identified patient data have been deposited at Mendeley Data and are publicly available as of the date of publication. The DOI is listed in the key resources table. This paper does not report original code. Any additional information required to reanalyze the data reported in this paper is available from the lead contact upon request.
