# Supplementary material for: Elevation of neural injury markers in patients with neurologic sequelae after hospitalization for SARS-CoV-2 infection
Source: iScience. 2022 Aug 1;25(8):104833. doi: 10.1016/j.isci.2022.104833 (PMC9341164; doi:10.1016/j.isci.2022.104833)

iScience, Volume *25*

Supplemental information

Elevation of neural injury markers in patients with neurologic sequelae after hospitalization for SARS-CoV-2 infection

Michail Spanos, Sigal Shachar, Thadryan Sweeney, H. Immo Lehmann, Priyanka Gokulnath, Guoping Li, George B. Sigal, Rajini Nagaraj, Pradeepthi Bathala, Farhan Rana, Ravi V. Shah, David A. Routenberg, and Saumya Das

Supplemental information

Elevation of Neural injury markers in Patients with Neurologic Sequelae after Hospitalization for SARS-CoV-2 Infection

Michail Spanos MD, Sigal Shachar PhD, Thadryan Sweeney MSc, H. Immo Lehmann MD, PhD, Priyanka Gokulnath PhD, Guoping Li PhD, George B. Sigal PhD,[2] Rajini Nagaraj PhD, Pradeepthi Bathala PhD, Farhan Rana BA, Ravi V Shah MD, David A. Routenberg PhD, and Saumya Das MD, PhD

Supplemental Figure 1. Levels of plasma Neurofilament Light Chain (NFL), Glial Fibrillary acidic Protein (Gfap) and Tau in pg/ml at admission comparing concentrations of analytes in plasma from hospitalized patients, outpatients and normal controls stratified by WHO score; related to figure 1.

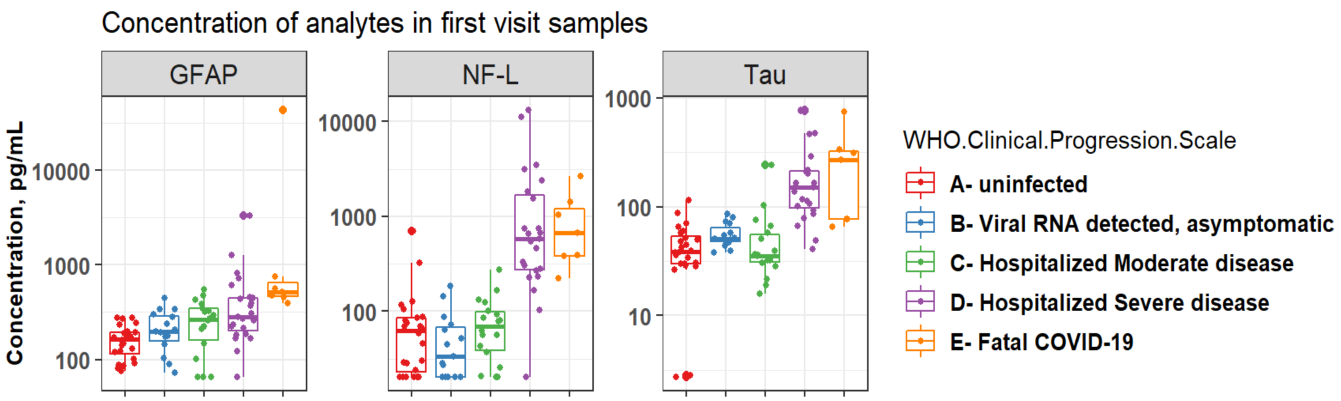

Supplemental Figure 2. Levels of plasma MCP-4 and TIM-3 in pg/ml at admission comparing concentrations of analytes in plasma from hospitalized patients, outpatients and normal controls stratified by WHO score; related to figure 1.

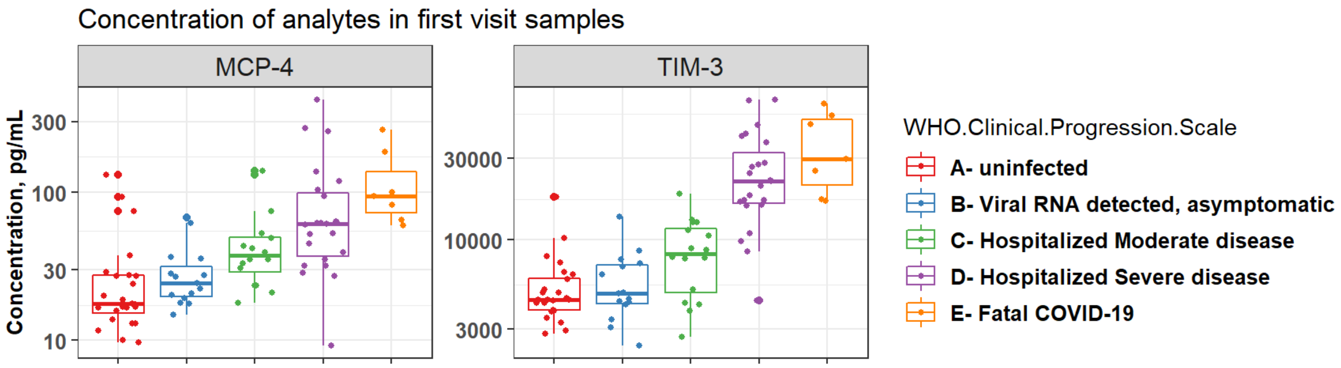

Supplement: Document S1. Figures S1 and S2 [file mmc1.pdf]
